# The cellular magnetic response and biocompatibility of biogenic zinc- and cobalt-doped magnetite nanoparticles

**DOI:** 10.1038/srep39922

**Published:** 2017-01-03

**Authors:** Sandhya Moise, Eva Céspedes, Dalibor Soukup, James M. Byrne, Alicia J. El Haj, Neil D. Telling

**Affiliations:** 1 School of Pharmacy, University of Nottingham, University Park, Nottingham NG7 2RD, United Kingdom; 2 Institute for Science and Technology in Medicine (ISTM), Keele University, Stoke-on-Trent ST4 7QB, United Kingdom; 3 IMDEA Nanociencia, Ciudad Universitaria de Cantoblanco, Madrid 28049, Spain; 4 Centre for Applied Geoscience (ZAG), University of Tuebingen, Tuebingen 72076, Germany

## Abstract

The magnetic moment and anisotropy of magnetite nanoparticles can be optimised by doping with transition metal cations, enabling their properties to be tuned for different biomedical applications. In this study, we assessed the suitability of bacterially synthesized zinc- and cobalt-doped magnetite nanoparticles for biomedical applications. To do this we measured cellular viability and activity in primary human bone marrow-derived mesenchymal stem cells and human osteosarcoma-derived cells. Using AC susceptibility we studied doping induced changes in the magnetic response of the nanoparticles both as stable aqueous suspensions and when associated with cells. Our findings show that the magnetic response of the particles was altered after cellular interaction with a reduction in their mobility. In particular, the strongest AC susceptibility signal measured *in vitro* was from cells containing high-moment zinc-doped particles, whilst no signal was observed in cells containing the high-anisotropy cobalt-doped particles. For both particle types we found that the moderate dopant levels required for optimum magnetic properties did not alter their cytotoxicity or affect osteogenic differentiation of the stem cells. Thus, despite the known cytotoxicity of cobalt and zinc ions, these results suggest that iron oxide nanoparticles can be doped to sufficiently tailor their magnetic properties without compromising cellular biocompatibility.

The ability of magnetic nanoparticles (MNPs) to transduce external magnetic field energy into a mechanical or thermal response can be exploited for biomedical applications, with research focussed on developing particles tailored to suit specific applications^1^,^2^,^3^,^4^,^5^,^6^,^7^. These particles’ magnetic response to an external magnetic field depends on properties such as their size, core composition and surface coating. Modifying their composition by doping transition metal cations into the iron oxides cores alters the nanoparticles’ magnetic moments^8^,^9^ and magnetic anisotropies^10^,^11^,^12^. By altering these two key properties the response of the nanoparticles to an external magnetic field can be defined. For instance, modifying the magnetic moment of the nanoparticles affects their efficiency as contrast agents in magnetic resonance imaging (MRI), whilst their magnetic anisotropy determines whether they are in a superparamagnetic state at physiological temperature (37 °C). In addition, both these properties affect the heating power of MNPs when exposed to high-frequency oscillating magnetic fields such as those used in magnetic hyperthermia^13^,^14^,^15^,^16^,^17^. This effect is currently being explored as a potential cancer therapy by using nanoparticles to deliver sufficient heating to trigger heat shock-associated cancer cell death^2^,^3^,^18^. Other applications utilising this heating property include heat-activated drug release using thermosensitive polymer coated nanoparticle carriers^4^,^19^, thermal imaging of target tissue^5^ and thermal activation of cell membrane ion channels^20^.

We have previously explored a bacterial synthesis route to obtain controlled biogenic preparation of magnetite nanoparticles, including those doped with either zinc or cobalt cations^21^,^22^,^23^,^24^. Analysis of these particles shows that they have a high degree of crystalline site ordering of the dopant cations^25^, leading to dramatic enhancements in either anisotropy in the case of cobalt dopants^23^,^24^ or magnetic moment for zinc-doped particles^21^. We have also assessed the magnetisation relaxation effects and heating properties of these doped particles, with relevance to magnetic hyperthermia applications, and found differences in heating efficiencies between zinc- and cobalt-doped particles that depend on their degree of mobility^26^. However before these properties can be further utilised in biological environments it is necessary to assess the effect of the introduction of transition metal ions on the biocompatibility of the iron oxide core.

Nanoparticles, when endocytically internalized by cells, are localized in lysosomes the highly acidic nature of which may corrode the core, releasing metal ions within the cell^27^,^28^. This is toxic to cells as metals such as zinc and cobalt, in their ionic form, are known cytotoxic agents^29^,^30^,^31^,^32^. Previous studies have shown evidence of cytotoxicity for chemically synthesized doped magnetite nanoparticles, indicating that doping modifies the biocompatibility of the nanoparticles^9^,^33^,^34^. Also, it is important to assess the effect doped MNPs have on normal cellular activities such as the ability of stem cells to differentiate along various lineages^6^,^35^,^36^,^37^, an important property being exploited in regenerative medicine therapies^38^,^39^,^40^,^41^.

In this work we assessed the suitability of doped magnetite nanoparticles for cellular applications, considering particles of the form M_x_Fe_3−x_O_4_, where M = Co (x = 0.4, 0.7, 1) or Zn (x = 0.4, 0.6, 0.9), obtained using the iron (III) reducing bacteria *Geobacter sulfurreducens*^21^,^22^,^23^,^24^. For comparison, we also examined undoped (zero cobalt or zinc doping) magnetite nanoparticles produced both biogenically and by traditional chemical synthesis (commercially available).

We explored the effect of nanoparticle association on two different cell types: primary human bone marrow derived mesenchymal stem cells (hMSCs), and human osteosarcoma derived cells (MG-63s). hMSCs are multipotent stem cells which have shown promise in treating connective tissue injuries^42^ such as osteoarthritis and various types of bone fractures. MNPs have been used in stem cell based therapies to trigger osteogenic differentiation as well as for stem cell tracking *in vivo*. Hence hMSCs are good candidates for evaluating the suitability of the doped MNPs for cellular applications. The MG-63s were also used as they mimic the bone precursor cell phenotype. Being a cancerous cell line, the comparison would help assess any associated changes in cytotoxic sensitivity to nanoparticles^43^ and give a better understanding of the doped MNPs interaction with different cells types.

As the changes in physiochemical conditions in a cellular environment could also affect the magnetic response of the nanoparticles^44^,^45^,^46^, we measured the AC susceptibility (ACS) of the MNPs^47^,^48^ to directly determine this response for doped biogenic and undoped chemically synthesized (synthetic) MNPs, both in free suspension and following cellular interaction.

ACS determines how the MNPs respond to an oscillatory (i.e. AC) magnetic field. When exposed to such a field the particles initially tend to partially align with the field direction. However, this magnetisation alignment can subsequently be relaxed either due to physical rotation of the particles caused by collisions with molecules in the suspending fluid (so called Brownian relaxation), or by the thermally induced reorientation of the internal particle magnetic spins (Neél relaxation). The shorter mode of relaxation is the more dominant one and this depends on various factors. For example the magnetic anisotropy and size of the MNP cores affect the Neél relaxation time, whilst environmental factors such as the viscosity of the solvent and temperature as well as the size of the MNP clusters (hydrodynamic size) affect the Brownian relaxation time. ACS is a complex quantity measured as a function of the frequency of the oscillating field, and is thus resolved into a real (*χ’*) and an imaginary (*χ”)* component. Whilst both components reflect the relaxation mechanisms that occur, a peak in the *χ”* component at a given frequency reveals the relaxation time for the particles. Generally, lower frequencies of the applied field match the Brownian relaxation times for particles which cannot rotate their internal magnetic spins (so called magnetically blocked particles). On the other hand, MNPs with small magnetic core sizes have shorter Neél relaxation times that match with higher field frequencies.

We have shown previously that ACS is an effective technique to non-invasively probe the magnetic response of nanoparticles in live cells^26^,^49^. In this study, we measured the ACS signal in cells associated with either zinc or cobalt-doped biogenic nanoparticles, as well as undoped nanoparticles. Further to this, we determined the cytotoxicity of these particles using differential live/dead cell staining, quantified by flow cytometry. Additionally, we studied the effect of cellular association with the nanoparticles on the osteogenic differentiation potential of hMSCs.

## Results

### Effect of cells on nanoparticles’ magnetic behaviour

Both zinc and cobalt doped biogenic MNP types have been thoroughly characterized by different techniques such as inductively coupled plasma analysis (ICP), X-ray diffraction (XRD), transmission electron microscopy (TEM) and X-ray absorption spectroscopy (XAS) as reported previously^21^,^24^. With doping, the core diameter of the nanoparticle decreases with increasing levels of cobalt or zinc as shown in Table 1. Core sizes are smaller than 15 nm with an average polydispersity of 0.3 for all the biogenic samples and are 24 nm for the synthetic magnetite sample^49^. The hydrodynamic diameter (cluster size) following citric acid coating of the nanoparticles are also shown. The sizes were calculated from ACS measurements for the nanoparticles in water using equation (1). The ACS technique has the advantage of measuring the hydrodynamic size of the particles based purely on their magnetic behaviour without interference by other non-magnetic^26^,^47^ material not associated with the nanoparticle and hence was preferred over the DLS technique when possible. However, due to the very weak magnetism of CoFe_2_O_4_^24^ and Zn_0.9_Fe_2.1_O_4_^21^, their hydrodynamic sizes cannot be measured via ACS and were hence obtained from dynamic light scattering (DLS) measurements. The sizes of the particle clusters range from 30–65 nm for most nanoparticles but are slightly larger (~85 nm) for the highest cobalt or zinc doping. However it should be noted that the final hydrodynamic size of the nanoparticle clusters is likely to be larger than the values given in Table 1, due to the effects of additional clustering in cell culture media and protein corona formation^49^,^50^.

**Figure 1:**
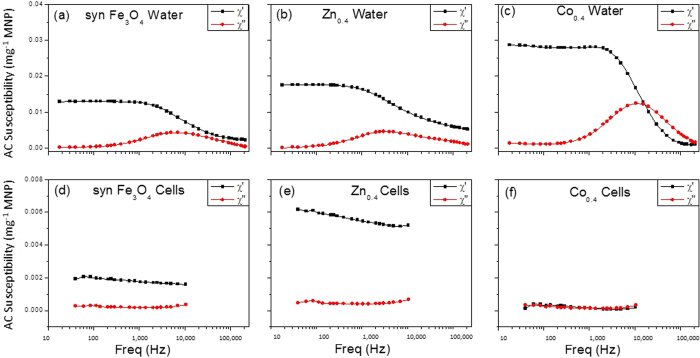
AC volumetric susceptibility curves of stable aqueous suspensions of nanoparticles and in cells: Measurements for synthetic Fe_3_O_4_ (**a**), Zn_0.4_Fe_2.6_O_4_ (**b**), and Co_0.4_Fe_2.6_O_4_ (**c**) in water and synthetic Fe_3_O_4_ (**d**), Zn_0.4_Fe_2.6_O_4_ (**e**), and Co_0.4_Fe_2.6_O_4_ (**f**) when associated with MG-63 cells following 72 hours incubation. [Data limited to lower frequencies in the more dilute samples (**d**–**f**)].

The ACS measurements for 100 μM solutions of synthetic Fe_3_O_4_, biogenic Zn_0.4_Fe_2.6_O_4_ and Co_0.4_Fe_2.6_O_4_ nanoparticles are compared in water and when incubated with cells (for 72 h) in Fig. 1. The peaks in the *χ”* curves seen for the aqueous suspensions of the three different MNP types (Fig. 1a–c) are typical of a Brownian magnetisation relaxation process, with the frequency position of this peak depending on the hydrodynamic size of the particles^26^. Although the nanoparticle core sizes vary between samples depending on the level of doping^21^,^24^, the similar positions of these χ” peaks in the ACS curves reveal comparable hydrodynamic particle/cluster sizes for all particle types (40–65 nm) (Table 1).

The *χ’* curves unlike the *χ”* curves show distinct variations in their relative shapes for the different particle types in water (Fig. 1a–c). For example, the cobalt-doped nanoparticles in water, show a symmetrical *χ”* peak at around 10 kHz (Fig. 1c) and a complete loss in the *χ’* signal beyond this value. This indicates a single dominating mode of relaxation which is expected to be Brownian relaxation for the magnetically blocked cobalt MNPs due to their high anisotropy^24^,^26^. In contrast, both undoped synthetic magnetite and zinc-doped nanoparticles have an asymmetrical *χ”* peak with the real (*χ’*) component greater than zero at higher frequencies. This can be explained by the presence of a fraction of nanoparticles with small core size, due to the polydisperse nature of the suspensions, exhibiting superparamagnetic properties and relaxing via the Neél relaxation mode to contribute to the ACS signal^26^.

In cells, the magnitude of AC susceptibility is reduced by a factor of ~20 for synthetic Fe_3_O_4_ (Fig. 1d) compared to a factor of ~10 for Zn_0.4_Fe_2.6_O_4_ (Fig. 1e). Most dramatically, the cobalt-doped MNPs, which showed the strongest signal in water, give a negligible response in cells with almost zero susceptibility signal (Fig. 1f). Further to this the peaks in the *χ”* component, associated with Brownian relaxation, are absent for all the particle types when in cells (Fig. 1d–f). These results are entirely consistent with the complete removal of the Brownian relaxation from the ACS signal such that only the weaker contribution from the MNPs relaxing via the Neél mode remains^26^.

### Effect of nanoparticles on cell viability

**Figure 2:**
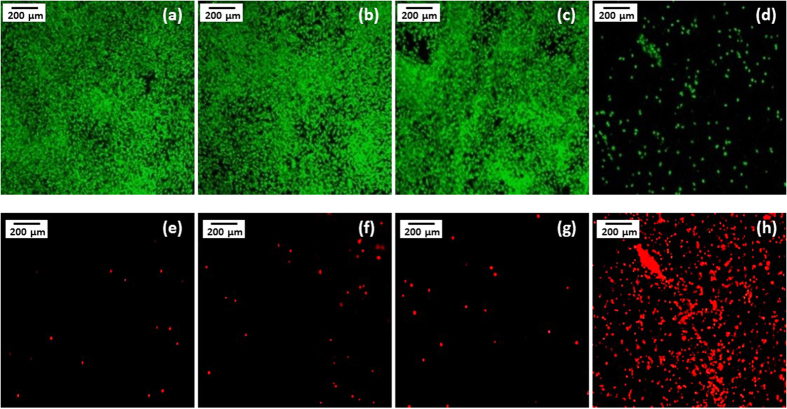
Cytotoxicity of 500 μM MNPs following 72 hours incubation with the human osteosarcoma cell line (MG-63s): Fluorescent micrographs of viable cells stained for Calcein (top) and compromised cells’ nuclei stained for ethidium homodimer (bottom). Cells not exposed to nanoparticles (**a**,**e**), exposed to synthetic Fe_3_O_4_ (**b**,**f**), CoFe_2_O_4_ (**c**,**g**) and CoCl_2_ (**d**,**h**) (scale bar 200 μm).

**Figure 3:**
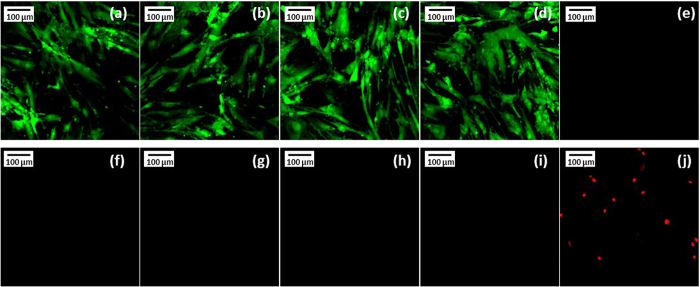
Cytotoxicity of 100 μM MNPs following 72 hours incubation on hMSCs: Fluorescent micrographs of viable cells stained for Calcein (top) and compromised cells’ nuclei stained for ethidium homodimer (bottom). Cells not exposed to nanoparticle (**a**,**f**), cells exposed to synthetic magnetite (**b**,**g**), Zn_0.4_Fe_2.6_O_4_ (**c**,**h**) Co_0.4_Fe_2.6_O_4_ (**d**,**i**) and CoCl_2_ (**e**,**j**) (scale bar 100 μm).

To further investigate the cellular interaction with the MNPs, we obtained fluorescent micrographs of human osteosarcoma MG-63 cell line (Fig. 2) and the primary hMSCs (Fig. 3) following treatment. The cells were incubated either with MNPs, or the equivalent concentration of known cytotoxic agent CoCl_2_^29^,^30^,^32^ salt to assess the relative cytotoxicity of the MNPs. This was achieved by using equivalent cation concentrations for CoCl_2_ and the MNPs, namely Co^2+^ for the salt and Co^2+^ + Fe^2+^ or Zn^2+^ + Fe^2+^ for the nanoparticles. These nanoparticles have already been shown to be internalized following 48 h incubation with MG-63 cells^49^.

**Figure 4:**
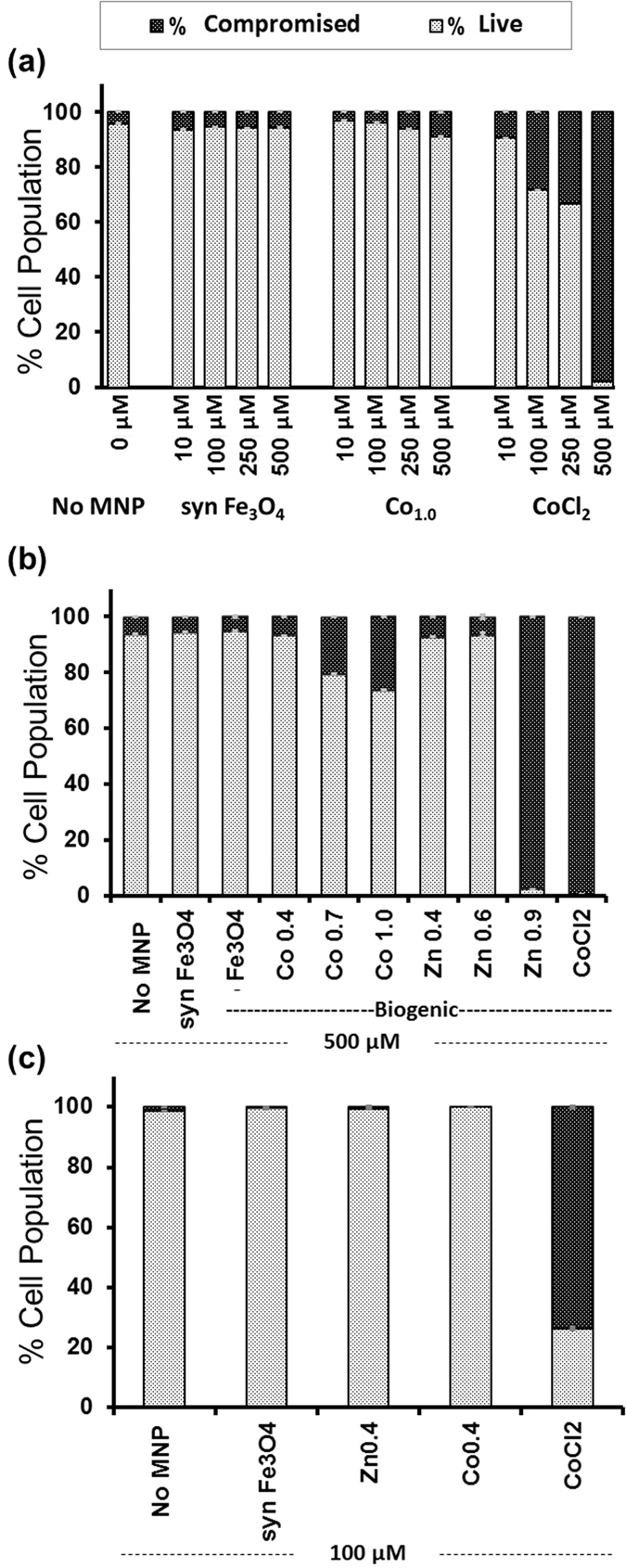
Cytotoxicity of MNPs following 72 hour incubation using flow cytometry quantification of live/dead staining: MNP incubation with human osteosarcoma (MG-63) cells using (**a**) varying concentrations of synthetic Fe_3_O_4_ (synFe_3_O_4_) and CoFe_2_O_4_ (Co1.0), and (**b**) 500 μM concentrations with varying dopant levels of Cobalt (0.4, 0.7, 1) and Zinc (0.4, 0.6, 0.9). (**c**) Cytotoxicity of Zn_0.4_Fe_2.6_O_4_ (Zn0.4) and Co_0.4_Fe_2.6_O_4_ (Co0.4) at 100 μM MNP concentrations in hMSCs. In all cases, the cytotoxic effect of equivalent concentrations of the Co^2+^ ion in its salt form (CoCl2) was compared to the metal nanoparticles. Columns represent the mean for n = 3 and the error bars the standard error of the mean. No error bars displayed for the following samples: (**a**) 250 μm, 500 μm CoCl_2_; (**b**) Zn_0.9_, CoCl_2_; (**c**) CoCl_2_.

In the case of the MG-63 cells, a large live population (stained green; Fig. 2a–c) and a correspondingly lower compromised population (stained red; Fig. 2e–g) is observed following exposure to synthetic Fe_3_O_4_ and the highest cobalt-doped MNP, CoFe_2_O_4_. Similarly, hMSCs exposed to synthetic Fe_3_O_4_, and biogenic Zn_0.4_Fe_2.6_O_4_ and Co_0.4_Fe_2.6_O_4_ have a large viable population (Fig. 3a–d) with minimal number of compromised cells (Fig. 3f–i). In contrast, a very large compromised population is observed for both cell types, when exposed to equivalent amounts of the ionic form of Co^2+^ as CoCl_2_ (Fig. 2d,h for MG-63s and Fig. 3e,j for hMSCs). These qualitative visual observations were backed up by quantitative flow cytometry to determine the percentage of cell populations staining for Calcein (live cells) and EthD (compromised cells), as shown in Fig. 4.

The effect of increasing the concentration of CoFe_2_O_4_ from 10 to 500 μM MNP concentration on MG-63 cells is compared to equivalent amounts of synthetic Fe_3_O_4_ and CoCl_2_ in Fig. 4a. Within the concentration range investigated, synthetic Fe_3_O_4_ is comparable to the no-nanoparticle control at all MNP concentrations with a ~95% live and ~5% compromised population. CoFe_2_O_4_ is also comparable to the no-nanoparticle control and synthetic magnetite, except at the highest concentration tested (500 μM). At this concentration of CoFe_2_O_4_, a minor increase in the compromised population is observed from 5% to ~10%. On the other hand, the CoCl_2_ treated cells show a dose dependant fall in the live population and a concomitant increase in the compromised population of up to 33% at 250 μM and an even higher loss of almost 98% at 500 μM Co^2+^ concentration.

The effect of the level of doping for cobalt and zinc was assessed in MG-63 cells at a fixed 500 μM MNP concentration, as shown in Fig. 4b. This high concentration was chosen as it is well above that required for applications, with significant cytotoxicity found for cells treated with the CoCl_2_ salt. The undoped synthetic and biogenic Fe_3_O_4_ MNPs had comparable biocompatibility to the nanoparticle-free control of almost 94% live population. For both zinc- and cobalt-doped MNPs, cytotoxicity increased in line with the level of doping, with the live population at 93% for Co_0.4_Fe_2.6_O_4,_ decreasing to 80% for Co_0.7_Fe_2.3_O_4_ and 74% for the highest cobalt doped sample of CoFe_2_O_4_. For zinc doping, the cytotoxic effect was more marked with an almost 98% compromised population for the highest zinc-doped MNP (Zn_0.9_Fe_2.1_O_4_). For the lower levels of zinc doping, namely Zn_0.4_Fe_2.6_O_4_ and Zn_0.6_Fe_2.4_O_4_ have a minor decrease in viability with a 93% live population for both particle types. As expected, the MG-63 cells treated with the equivalent CoCl_2_ concentration, showed almost 100% loss in viability (~100% compromised cells).

We found that hMSCs were more sensitive to the addition of cytotoxic agents, with a ~70% compromised population when treated with only 100 μM CoCl_2_ (Fig. 4c). For this reason, to make comparative cytotoxic measurements using MNPs, the hMSCs were also treated with 100 μM concentrations of synthetic and doped biogenic MNPs (Fig. 4c), setting cobalt and zinc doping levels consistent with those required to induce the enhanced magnetic properties discussed earlier. In these cases the cellular viability at 100 μM of synthetic magnetite, Co_0.4_Fe_2.6_O_4_ and Zn_0.4_Fe_2.6_O_4_ was at 99% live population and comparable to the untreated population, showing negligible evidence of a cytotoxic response (Fig. 4c).

**Figure 5:**
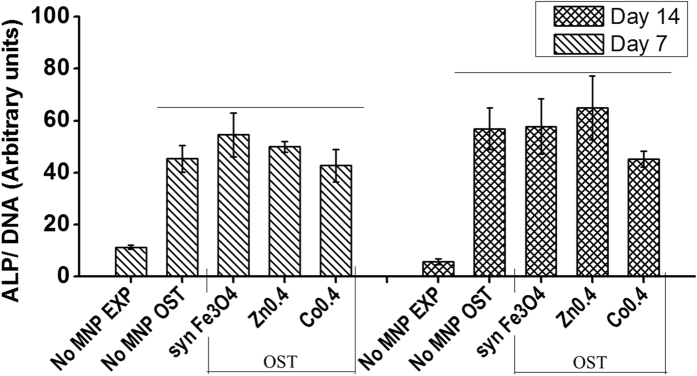
Effect of MNPs on the osteogenic differentiation potential of hMSCs: hMSCs were exposed to 100 μM of synthetic Fe_3_O_4_ (syn Fe_3_O_4_), Zn_0.4_Fe_2.6_O_4_ (Zn0.4) and Co_0.4_Fe_2.6_O_4_ (Co0.4) for 72 hours following which they were incubated in osteogenic (OST) media. Osteogenesis was measured through ALP production and normalized to cell number by measuring DNA content at day 7 and 14. ALP production was compared to cells not exposed to nanoparticles grown in expansion media (No MNP EXP) and osteogenic media (No MNP OST). Columns represent the mean for n = 3 and the error bars the standard error of the mean. Line over columns indicates groups that were not significantly different from each other (Tukey’s HSD, p < 0.05).

Following cytotoxicity studies, the doped MNPs were assessed for possible effects on the differentiation potential of hMSCs. The osteogenic differentiation of hMSCs as measured via the alkaline phosphatase (ALP) assay^51^ at day 7 and 14 is shown in Fig. 5. Compared to the cells cultured in expansion media, cells cultured in osteogenic differentiation media show a significant up-regulation in the ALP production at day 7 and day 14^52^. However the ALP production of cells following internalization of synthetic Fe_3_O_4_, Zn_0.4_Fe_2.6_O_4_ and Co_0.4_Fe_2.6_O_4,_ in osteogenic differentiation media, is not significantly different from the control cells in the same media (Fig. 5).

## Discussion

In this study we explored the effect of interactions of cells and nanoparticles on the behaviour of each other. By comparing undoped, zinc and cobalt-doped iron oxide nanoparticles with differing magnetic properties we were able to highlight the changes in magnetic behaviour, especially in terms of the relaxation mechanisms when the MNP’s were associated with cells. The AC susceptibility measurements showed that the Brownian relaxation component observed for all the nanoparticle types when as aqueous suspensions was lost when they were associated with cells. This could be due to immobilization of the nanoparticles when in cells which in turn could be because the nanoparticles were bound to the plasma membrane or intracellular membranes of cells. This result matches with the findings in the study by Hilger *et al*.^53^, where magnetorelaxometry measurements confirmed presence of MNPs relaxing via the Néel mode immobilized in cells. On the other hand, they did not study the effect on the Brownian relaxation mode of the MNPs. Another possible explanation for the loss in the Brownian mode of relaxation could be the aggregation of the nanoparticles upon internalization. The larger clusters could have sizes greater than that detectable by our ACS instrument’s frequency range (the maximum hydrodynamic size detectable in water is ~350 nm).

The relatively strong ACS signal from the zinc-doped nanoparticles in cells, compared to the cobalt-doped particles, has implications for intracellular magnetic heating applications that exploit magnetisation relaxation mechanisms. Previously we found that the comparatively strong magnetic hyperthermia effect measured in aqueous suspensions prepared from similar cobalt-doped nanoparticles, was suppressed following their immobilisation in glycerol^26^. A similar loss of the Brownian component when MNPs are associated with cells implies their heating efficiency will be suppressed unless strong magnetic field amplitudes are applied. Conversely the survival of Néel relaxation for the zinc-doped nanoparticles associated with cells, suggests cellular based magnetic hyperthermia might be possible with these particles even under clinically relevant magnetic field conditions.

Apart from the effect cells have on the magnetic behaviour of nanoparticles, the nanoparticles in turn could affect cellular functioning. Especially with the introduction of zinc and cobalt, changes in cytotoxicity could occur due to leaching of the metal ions from the nanoparticle core. By comparing to equivalent levels of free Co^2+^ ions, we find that the nanoparticles have lower levels of toxicity for the same cation concentrations. While the CoCl_2_ salt forms Co^2+^ ions in solution quite rapidly and will have an immediate effect on cell viability, the citric acid surface coating of the magnetic nanoparticles delays leaching of the metal core and hence release of toxic ions into solution. Indeed, uncoated ~30 nm cobalt nanoparticles at concentrations as low as 2 μM have previously been shown to significantly reduce the viability of human fibroblasts within 72 h of exposure^27^. In addition, the cations are tightly bound within a magnetic lattice^21^,^23^,^24^ which reduces release of the metal ions into solution. With time, when the nanoparticles are internalized by cells, they are stored within lysosomes^6^ the acidic environments of which could cause leaching of the surface coating and subsequent release of the core metal ions. This could explain the increase in cytotoxicity with increasing level of doping observed in our study. This effect was also observed in another study^54^ evaluating the cytotoxicity of chemically synthesized zinc-doped iron oxide nanoparticles. A dose dependant decrease in mitochondrial metabolic activity (as measured via MTT) and increase in membrane damage (as measured via the LDH assay) was observed in human lung epithelial (A549), skin epithelial (A431) and liver (HepG2) cells. An almost 60% reduction in the mitochondrial metabolic activity and ~60% increase in membrane damage was observed for all three cell types following 24 h exposure to 40 μg/ml of the uncoated MNPs.

Another parameter that has been shown to affect nanoparticle cytotoxicity is their size. A study on uncoated silver nanoparticles showed that cytotoxicity increased with decrease in the core size of the nanoparticles^55^ from >100 nm to 20 nm. This is attributed to their large surface-to volume ratio resulting in faster dissolution of the core with higher rate of ion release^56^ thereby affecting cell viability. In the nanoparticles that we investigated, increasing levels of doping causes a corresponding decrease in core sizes. The increase in cytotoxicity of the nanoparticles with increase in level of doping observed in this study could be a combined contribution of the increasing levels of dopant and the decrease in the nanoparticle core size.

When comparing the cytotoxicity of the doped MNPs, a more pronounced increase in cytotoxicity with level of doping was found with zinc than cobalt which shows a similar but much more gradual trend. The highest zinc-doped MNP (Zn_0.9_Fe_2.1_O_4_) heavily affects cellular viability (with an almost 99% compromised population). One possible explanation for this could be because zinc ions are more cytotoxic than cobalt ions^31^. However, another possible explanation could be the interaction of the zinc with the citrate surface coating. It is known that the solubility of zinc oxide is enhanced in the presence of citrate and that it depends on the core nanoparticle size irrespective of the cluster (hydrodynamic) sizes^57^. This, together with the higher levels of zinc doping, would explain the enhanced toxicity as the Zn_0.9_Fe_2.1_O_4_ MNPs which have smaller core sizes and hence higher surface area in comparison to the lower doped zinc nanoparticles^21^,^24^. On the other hand, cobalt oxide solubility is not significantly affected by presence of citrate^58^ and hence a gradual and minor increase in cytotoxicity is observed with increasing levels of cobalt doping.

Apart from cell viability the differentiation behaviour of stem cells was also investigated in this study. The osteogenic differentiation of the mesenchymal stem cells was found to be unaffected by their uptake of the zinc and cobalt-doped nanoparticles as measured via the ALP marker expression. More generally, the effect of magnetic nanoparticles on the trilineage differentiation of stem cells seems to depend on the particle type and the lineage of differentiation. For instance while chitosan-coated superparamagnetic iron oxide particles did not interfere with osteo-/adipo-/ chondrogenic differentiation of bone marrow derived hMSCs^59^, clinically approved MRI contrast agent nanoparticle Resovist/Ferucarbotran was shown to affect differentiation^60^,^61^. While one study shows that Resovist particles inhibit only the chondrogenic differentiation^60^ (not adipo- or osteogenic) another study^61^ shows that the same nanoparticle does inhibit osteogenic differentiation in the same cell type. Such contradicting results increase the need to thoroughly assess effects of all different nanoparticles on stem cell behaviour. Further studies are needed to ensure that the zinc- and cobalt-doped nanoparticles used in this study do not affect the mineralization, adipogenic and chondrogenic potential of hMSCs. The results obtained here identify the potential of these biogenic doped magnetite nanoparticles for cellular applications.

In conclusion, our results show that biogenic nanoparticles with moderate levels of cobalt and zinc doping, with optimised magnetic properties for cellular applications, have minimal short term acute cytotoxicity within concentrations suitable for cellular applications. In addition preliminary studies show that they that do not interfere with osteogenic differentiation. The assessment of their magnetic behaviour when associated with cells shows a diminished magnetic response depending on the particle type, indicative of reduced particle mobility. Particularly, a strong Néel magnetisation relaxation mechanism is preserved in zinc-doped iron oxide nanoparticles which is suitable for applying localised and controlled heating effects in cellular hyperthermia. However further studies monitoring the longer term toxicity and fate *in vivo* are needed to assess the full clinical potential of these particles.

## Methods

### Magnetic nanoparticle suspensions

Chemically synthesized magnetite (referred to as synthetic magnetite) was obtained from Sigma- Aldrich, UK. The biogenic nanoparticles were produced via microbial iron reduction of zinc and cobalt-doped ferrihydrite by the iron reducing bacteria *Geobacter sulfurreducens* according to the methods described previously^21^,^24^.

Stable aqueous suspensions of nanoparticles were prepared by citric acid coating using procedures described in detail elsewhere^13^,^24^. The suspensions were sterilized using 0.2 μm filters prior to use in cellular experiments. Nanoparticle suspension concentrations were measured using the Ferrozine method for iron following complete digestion by concentrated nitric acid (70%) at high temperatures (>60 °C) overnight.

Nanoparticle sizes were characterized using dynamic light scattering (DLS) measurements as aqueous suspensions in a Zetasizer 3000 (Malvern, UK) instrument.

### Cell culture

MG-63, an osteosarcoma cell line (Lonza, UK) and human bone marrow derived primary mesenchymal stem cells (less than 5 passages) (Lonza, UK) were seeded in expansion media consisting of 4.5 gL^−1^ glucose Dulbecco’s Modified Eagle’s medium (Lonza, UK) supplemented with 10% foetal bovine serum, 1% Penicillin/Streptomycin (antibiotics and antimycotics) and 1% L-glutamine in well plates at ~80% confluency and allowed to attach overnight before addition of nanoparticle suspensions. hMSCs were adherence selected and tested for stemness using standard trilineage differentiation and flow cytometry for CD markers. Aqueous suspensions of the nanoparticles were mixed with cell culture media at 10% v/v and then added to cells. For the control samples, equivalent amount of sterile distilled water (negative control) or sterile cobalt chloride solution (positive control; Sigma- Aldrich, UK) at a concentration of 500 μM were mixed with the cell culture media and added to cells. The cation/MNP concentrations were maintained constant between comparisons and cells were incubated with the additives for 72 h before being assessed.

### AC Magnetic Susceptibility

The AC magnetic susceptibility was measured in a custom built AC susceptometer on 200 μl of the sample contained in a glass vial. The oscillating magnetic field frequency was swept from 10 to 210 kHz during measurement. For measurements of MNPs associated with the cells, cells were incubated with MNPs for 72 h following which they were trypsinized. The trypsinized cells were resuspended in fresh media and transferred to the glass vial for AC susceptibility measurements. The cell samples had low levels of magnetic material which fell below the detection limits of AC susceptometer at the higher frequency range. Hence, measurements for these samples were limited to <10 kHz frequency. All susceptibility measurements were performed at 37 °C (310 K) to maintain physiological temperature.

For Brownian relaxation, the hydrodynamic radius r_H_, can be calculated using the frequency (f_B_) of the *χ”* peak, from equation (1) where *V*_*H*_ is the hydrodynamic volume (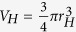), *η* the viscosity of the solvent (water, *η* = 1 × 10^−3^ Pa.s), *k*, the Boltzmann constant (*k* = 1.38 × 10^−23 ^kgms^−2^ K^−1^) and *T*, the temperature (K). The frequency limits of 10 Hz and 210 kHz mean that MNPs with hydrodynamic diameter between ~350 to ~13 nm can be detected when suspended in water at 37 °C (310 K).


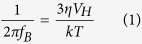


### Microscopy

The live dead dual staining kit for mammalian cells (Life Technologies, UK) was used to assess cell viability. 2 μM of Calcein-AM (CAM) in phosphate buffered saline (PBS) was added to stain live cells green and 4 μM of the nuclear stain ethidium homodimer (EthD) in PBS was added to stain compromised cells red. Cells were incubated with the dyes at 25 °C in the dark for 30 min. Following this, excess stain was washed off and fresh media added. Cells were immediately imaged under bright field and fluorescent conditions in Olympus IX83 confocal microscope fitted with a camera using the Fluorview 10 software.

### Flow cytometry quantification

For flow cytometry, cells were seeded in 24 well plates and tested similar to the fluorimetry samples. Staining was performed using 1 μM CAM and 2 μM EthD as these concentrations gave optimum levels of fluorescence in the cytometer. Incubation with the dyes was performed at 37 °C for 15 min. Incubation at higher temperatures caused the cells to lift off compared to incubation at room temperature and this helped obviate the trypsinization step during sample preparation circumventing any deleterious effect trypsin might have on cell viability. The cells were resuspended in round-bottom tubes, washed and resuspended in flow cytometry buffer (1% bovine serum albumin in PBS). Five to ten thousand events gated on size (forward scatter) and granularity (side scatter) were analysed. For flow cytometry, a separate set of controls were prepared for every set of samples. The controls included live cells without any staining, stained only for CAM, only for EthD and for both the dyes. Identical set of controls were prepared for compromised cells (cells treated with 70% methanol). The controls were used for setting the parameters and compensation values in the flow cytometer.

Flow cytometry was performed on a Becton Dickinson FC500 flow cytometer The CellQuestPro software (Becton Dickinson, UK) was used for data acquisition and the Cyflogic software (CyFlo Ltd.) for further data analysis. CAM fluorescence was measured using the FL1 gate in the flow cytometer while EthD fluorescence was measured in the FL3 region. Compensation for leakage of CAM into FL3 region was performed by matching the geometric mean of unstained cells’ fluorescence in FL3 to that of live cells stained for only CAM. Similar compensation was performed for leakage of EthD fluorescence in the FL1 using unstained and compromised cells stained for EthD alone. 99% of the unstained cell population was set to fall under the live staining background using FL1 histogram and similarly for the compromised staining background values. These setting were then used to filter the background fluorescence for the actual samples in FL1 vs. FL3 log plot.

### Osteogenic differentiation studies

Cells were seeded at 12,000 cells/well in 24 well plates and incubated with MNPs for 72 h. Following this, the unbound MNPs were washed off with PBS and expansion media or osteogenic media were added. Osteogenic differentiation media was prepared by adding 0.1 μM Dexamethasone, 10 mM β-glycerophosphate, 50 μM ascorbic acid and 1% non-essential amino acids (all from Sigma- Aldrich, UK) to the expansion media. Media was replaced every 3 days once and at day 7 and 14, wells were washed with PBS and sterile water was added to wells. Cells were lysed by repeated freeze-thaw cycles. Lysates were used for both ALP and DNA assays. ALP was measured via the 4-Methylumbelliferyl phosphate (4-MUP) Liquid Substrate System (Sigma- Aldrich, UK). In brief, lysates were incubated with equal volume of 4-MUP reagent for 30 min in the dark at 37 °C. Following this, the fluorescence was measured in a plate reader (BioTek Instrument Inc.) at Ex/Em of 360/440 nm. For normalizing to the number of cells, the double stranded DNA content was measured using the Quant-iT™ PicoGreen^®^ dsDNA Assay Kit (Life Technologies, UK). Lysates were incubated with 1.5 times volume of the Picogreen reagent at room temperature in the dark for 15 min. After incubation, the fluorescence was read at Ex/Em of 485/535 nm.

### Statistics

Each column in Figs 4 and 5 represent the mean (n = 3) and the error bars are the standard error of the mean. For flow cytometry (Fig. 4), experimental triplicates (n = 3) were assessed with each sample measured until 10,000 counts were obtained. In some samples with very low viability, due to insufficient cell numbers, the triplicates were combined to form a single sample and measured to obtain 10,000 counts. Hence for these samples, no error bars are shown in the columns and because of this it was not possible to perform a statistical comparison for significance in the data in this figure. For Fig. 5, one-way ANOVA was performed in conjunction with Tukey’s HSD test. The sample variance was confirmed to be equal between groups for ANOVA.

## Additional Information

**How to cite this article**: Moise, S. *et al*. The cellular magnetic response and biocompatibility of biogenic zinc- and cobalt-doped magnetite nanoparticles. *Sci. Rep.* **7**, 39922; doi: 10.1038/srep39922 (2017).

**Publisher's note:** Springer Nature remains neutral with regard to jurisdictional claims in published maps and institutional affiliations.

## Figures and Tables

**Table 1:** Core and hydrodynamic sizes of the nanoparticles: Core sizes as measured via TEM^21^,^24^ and hydrodynamic sizes of aqueous suspensions of citric acid-coated MNPs as calculated from ACS measurements or in the case of CoFe_2_O_4_ and Zn_0.9_Fe_2.1_O_4_ from DLS measurements.

Particle type	Core diameter (nm)	Hydrodynamic diameter (nm)
Synthetic Fe_3_O_4_	24	48
Biogenic Fe_3_O_4_	16	37
Co_0.4_Fe_2.6_O_4_	13	42
Co_0.7_Fe_2.3_O_4_	8	34
CoFeO_4_	2–4	84
Zn_0.4_Fe_2.6_O_4_	11	64
Zn_0.6_Fe_2.4_O_4_	11	37
Zn_0.9_Fe_2.1_O_4_	8	87
